# The Changing Landscape of Academic Anesthesiology Research Funding: Preliminary Insights from a National Survey

**DOI:** 10.7759/cureus.103599

**Published:** 2026-02-14

**Authors:** Shahla Siddiqui, Ashish Khanna, Avery Tung, Olivier Duranteau, Ronald Pearl, Alexander Nagrebetsky, Michael O'Connor, Jarva Chow, Ranjit Deshpande, Megan Hicks, Martin Krause, Vanessa Moll, Ricardo E Verdiner, Kunal Karamchandani, Justin Tawil, Dolores Njoku, Gyorgy Frendl

**Affiliations:** 1 Anesthesia, Beth Israel Deaconess Medical Center (BIDMC), Boston, USA; 2 Anesthesia, Wake Forest School of Medicine, Charlotte, USA; 3 Anesthesia, UChicago (The University of Chicago), Chicago, USA; 4 Anesthesia, Hôpital d'Instruction des Armées Percy, Paris, FRA; 5 Anesthesia, Stanford University, Palo Alto, USA; 6 Anesthesia, Massachusetts General Hospital, Boston, USA; 7 Anesthesiology and Critical Care, Yale School of Medicine, New Haven, USA; 8 Anesthesia, Wake Forest Baptist Medical Center, Charlotte, USA; 9 Anesthesia, University of California San Diego, San Diego, USA; 10 Anesthesiology, University of Minnesota, Minneapolis, USA; 11 Critical Care Medicine, University of Minnesota, Minneapolis, USA; 12 Anesthesiology and Critical Care, Mayo Clinic Alix School of Medicine, Rochester, USA; 13 Anesthesiology, University of Texas Southwestern Medical Center, Dallas, USA; 14 Anesthesia, University of Wisconsin, Madison, USA; 15 Anesthesia, Washington University School of Medicine, St. Louis, USA; 16 Anesthesia, Brigham and Women's Hospital, Boston, USA

**Keywords:** academic medicine, burnout, federal funding, progress, research

## Abstract

This national survey of academic anesthesiology researchers offers an important and timely window into the evolving landscape of scholarly activity within our specialty. Over the past decade, anesthesiology has experienced a convergence of stressors--declining federal and foundation funding, inconsistent institutional investment in research infrastructure, shifting scientific priorities, and escalating clinical demands--that collectively threaten the stability and future growth of the academic workforce. Over 50% of the anesthesiology workforce is over 55 years of age. Many of these physicians are approaching retirement, and the anesthesiology workforce is shrinking relative to clinical need and demand. This places enormous pressure on Academic Medical Centers (AMCs) to retain and recruit faculty to not only engage in research but also teach the next generation of anesthesiologists. The preliminary survey findings reflect a community that is deeply committed to advancing scientific inquiry but increasingly strained by structural and systemic challenges that impede productivity, innovation, and career development.

## Introduction

Academic anesthesiology is undergoing substantial transformation as researchers navigate shrinking funding streams, increasing clinical obligations, and shifting institutional priorities [1]. The recent changes in academic medicine caused by a shift in federal funding priorities stem primarily from major funding cuts, changes to indirect cost reimbursement, and the cancellation of grants related to humanities, diversity, equity, and social sciences [2]. To better understand how these pressures impact the anesthesiology research workforce, we conducted a national survey assessing funding stability, research experiences, institutional support, and professional well-being among faculty across the United States.

## Materials and methods

This anonymous survey (Appendices) was developed and validated by the authors after iterative analysis and feedback. Face and content validity were established through pilot testing with experts. A validated stress response tool (Perceived Stress Scale or PSS) was also included to determine levels of stress around the present funding environment on academic anesthesiologists who are members of two anesthesiology societies [3]. The survey was distributed among the members of the Committee on Critical Care Medicine of the American Society of Anesthesiologists (ASA) [4], as well as the Association of University Anesthesiologists (AUA) [5] via a REDCap link. Simple frequency statistics, as well as qualitative analyses, were used with the assistance of AI to generate the main results. The PSS is a widely used psychological instrument designed to measure an individual's perception of stress in their life. The PSS is a widely validated instrument with strong internal consistency (Cronbach α ≈ 0.78-0.91) and is commonly used in physician burnout and well-being research. The PSS-10 measures the degree to which situations in life are appraised as stressful, with total scores ranging from 0 to 40. Interpretation typically categorizes scores as 0-13 (low stress), 14-26 (moderate stress), and 27-40 (high perceived stress). Higher scores indicate higher levels of perceived stress and lower self-efficacy.

To ensure methodological rigor, the survey instrument underwent multiple stages of refinement, beginning with item generation informed by a review of the literature on academic workforce challenges, burnout, and research infrastructure in anesthesiology and other procedural specialties. Draft items were iteratively piloted among a small group of academic anesthesiologists with expertise in critical care, medical education, and research methodology. Feedback was solicited on clarity, relevance, comprehensiveness, and overall burden. Items were revised to reduce ambiguity, eliminate redundancy, and ensure that constructs such as institutional support, research time, mentorship availability, and perceptions of the funding climate were captured in a multidimensional and interpretable manner.

The final survey consisted of structured multiple-choice questions, Likert-scale items, and several open-ended free-text fields that allowed respondents to elaborate on emerging challenges or propose specific recommendations. This hybrid design enabled both quantitative characterization of trends and rich qualitative insights to contextualize and deepen the interpretation of numerical findings. The inclusion of the PSS provided a validated and standardized mechanism to assess stress levels that could be compared to normative data from physician and non-physician populations. Given the increasing relevance of burnout, emotional exhaustion, and moral distress in academic anesthesiology, particularly amid rising clinical demands and shrinking research resources, the addition of a validated psychometric tool ensured that self-reported concerns were not interpreted in isolation from broader indicators of well-being.

The survey links were disseminated through professional society listservs and leadership channels to maximize distribution across diverse academic institutions, including large research-intensive medical centers, hybrid clinical-academic departments, and smaller programs with limited research infrastructure. Respondents represented a broad spectrum of academic ranks, ranging from junior faculty to full professors, as well as individuals with leadership roles such as division chiefs, vice chairs of research, and fellowship directors. While the sample size reflects the inherent challenges of surveying busy clinician-scientists, particularly in a specialty with variable levels of research engagement, the population sampled is highly enriched for individuals directly affected by national research trends, NIH funding fluctuations, and institutional resource allocation.

Quantitative data were analyzed using simple descriptive statistics (frequencies, proportions, and mean values), appropriate to the exploratory nature of the project. These statistics were complemented by thematic qualitative analysis of free-text responses. Microsoft Co-Pilot AI-assisted clustering and natural language processing techniques were used to support coding, aggregation of themes, and identification of recurrent patterns. All AI outputs were manually reviewed by the authors to ensure accuracy, eliminate misclassification, and preserve interpretive nuance. Themes were further refined using a consensus discussion, ensuring reliability and internal validity of the qualitative synthesis.

Ethical considerations were addressed through anonymization of all responses and removal of identifiable institutional characteristics. Participation was voluntary, with implied consent through survey completion. Because the project was classified as minimal-risk quality improvement/needs assessment in alignment with ASA committee work, a formal IRB review exemption was obtained from BIDMC CCI (2025D000490 Version: 1).

Overall, the methodological approach allowed for a comprehensive and multidimensional assessment of contemporary challenges facing academic anesthesiology researchers. By integrating validated measures, such as the PSS, with both structured and open-ended survey items, the study captures both quantitative trends and the lived experiences of academic anesthesiologists navigating an increasingly strained research ecosystem. The resulting dataset provides a robust foundation for interpreting the current landscape, identifying emerging vulnerabilities within the academic workforce, and informing recommendations for sustaining and strengthening the future of anesthesiology research.

## Results

Respondent characteristics

Well-Being and Stress

The survey included 44 respondents from 20 U.S. states, representing junior (5 assistant professor ranks), mid-career (10 associate professor ranks), and senior academic anesthesiologists (29 full professors with 8 having tenure) (Table 1). The response rate was 6.8%. Thirty-three (75%) respondents worked in a university-affiliated hospital, 7 (16%) in a private academic institution, and the remaining in a public academic one. Of these, 57% had been associated with academics for more than 20 years, with 61% holding NIH funding.

**Table 1:** Survey overview

Total respondents	44
States represented	20
Complete responses	42
Incomplete responses	2
Multi-institutional study participation	32 respondents

Funding Landscape

Participants reported involvement in independent or collaborative research, with 32 respondents taking part in multicenter studies and nearly half serving as site or study principal investigators. A notable subset of respondents (12) reported reductions in research funding, describing abrupt departmental cuts, shrinking pilot award programs, and uncertainty around federal mechanisms such as NIH and AHRQ [6]. Eleven respondents noted stalled or unpredictable award cycles that disrupted ongoing research and threatened the continuity of lab personnel and infrastructure. Despite funding challenges, most respondents (31) did not change their research focus (Table 2).

**Table 2:** Impact of funding changes DEI: diversity, equity, and inclusion

Respondents reporting funding reductions	12 (27%)
Institutions with local pilot grants	Varied
Shifts in research focus	10 respondents
Perceived decline in DEI research support	Frequent comment (lack of DEI research)

Career Trajectories and Pressures

**Figure 1:**
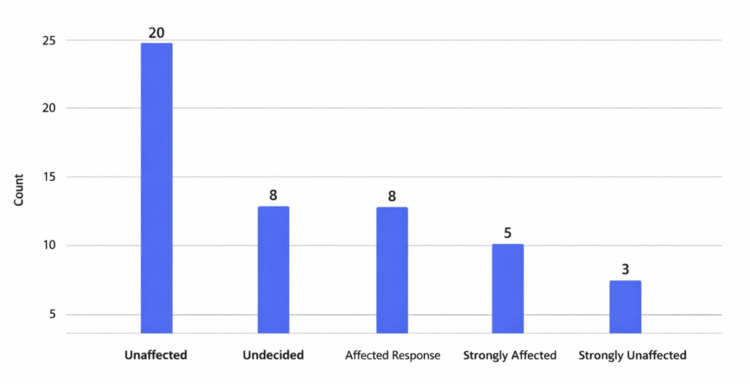
Number of participants affected by funding changes

Thirty-six respondents reported no change in core facilities and mentor support, and the remaining reported a slightly less supportive environment. While most respondents maintained their research focus, 10 shifted direction due to funding limitations or shifts in institutional and national priorities. Respondents expressed particular concern about declining support for health equity and diversity, equity, and inclusion (DEI)-focused research, reflecting broader social changes. Thirteen respondents reported an increase in clinical duties, with most reporting no change. Some (15) reported an increase in layoffs of research-related staff directly because of the funding budget cuts (Figure 1).

Well-Being, Stress, and Burnout

**Figure 2:**
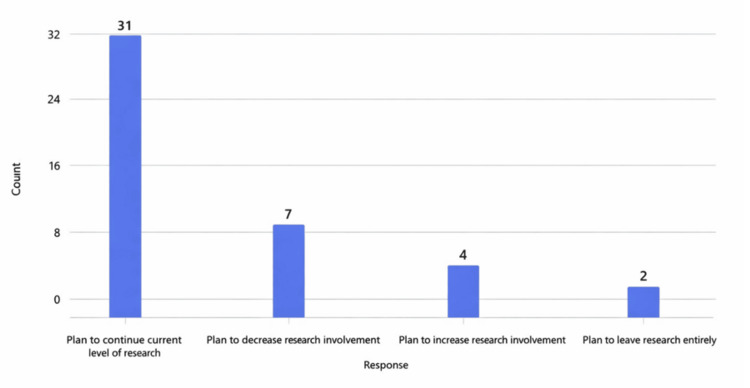
Participants' research plans after funding changes

Although the majority (32) of the respondents did not feel an impact on their academic promotion or career advancement, 27 reported elevated stress, reduced sense of control, and frustration tied to poor job satisfaction due to funding instability and increased clinical demands. Sixteen reported feeling burnout symptoms, with five strongly affected. Despite these pressures, many retained confidence in their problem-solving abilities and resilience, although some reported considering transitions to industry or early retirement. The average PSS for negative experiences was 15.3/40, and the average positive experience score was 27.9/40 (Table 3). Nine of the academic anesthesiologists who responded planned to decrease or leave research altogether due to the changing environment and challenges (Figure 2).

**Table 3:** Perceived Stress Scale (0-40)

Negative Experiences	Been upset because of something that happened unexpectedly? 15.6; Felt that you were unable to control the important things in your life? 16.7; Felt nervous and 'stressed'? 17.7; Found that you could not cope with all the things that you had to do? 12.1; Been angered because of things that were outside of your control? 17.2; Felt difficulties were piling up so high that you could not overcome them? 12.6
Positive Experiences	Felt confident about your ability to handle your personal problems? 32.3; Felt that things were going your way? 25.8; Been able to control irritations in your life? 27.7; Felt that you were on top of things? 25.8

## Discussion

In this small survey of a sample of academic anesthesiologists from two societies, funding instability was widespread, with many respondents reporting reduced awards, disrupted funding cycles, and departmental cuts that threatened research continuity. Increased clinical demands, shifting institutional priorities, and erosion of support for equity-focused research contributed to stress, burnout, and career uncertainty. Despite maintaining productivity and research focus, many described worsening job satisfaction, loss of research staff, and growing pressure to shift toward clinically driven or industry-based work. Notably, one in five respondents planned to reduce or exit research altogether, raising concerns about the sustainability of the academic anesthesiology pipeline.

Consequences for the workforce and academic missions

Our survey is substantially limited by its 6.8% response rate (n=44), which critically constrains the generalizability of the findings and increases the likelihood that results may not reflect the broader population of academic anesthesiologists. The use of ASA and AUA listservs as the sole recruitment channels introduces additional selection bias, as these platforms may overrepresent individuals already engaged in national societies and underrepresent those less connected to academic networks. Moreover, there is significant potential for non-response bias, with responses likely skewed toward two ends of the spectrum: those experiencing the greatest distress, who felt compelled to share their challenges, and those with greater academic security or protected time who had the capacity to complete the survey. Finally, the small sample size provides insufficient statistical power for meaningful subgroup analyses, limiting our ability to detect differences across rank, institution type, funding status, or demographic groups. Together, these limitations significantly narrow the interpretability of our findings and should temper any broad inferences.

Our preliminary data, while not representative, suggest potential risk [7]. Growing dependence on clinical revenue and productivity-based compensation models poses an impending crisis in sustaining meaningful academic output [8]. Several individuals are considering leaving or have already left academic medicine early. These observations are consistent with broader national trends where academics are transitioning to industry for better-paid opportunities [9]. Pay increases for clinical work over the academic mission of teaching and research are an existential threat to academic medicine. Additionally, institutions must recognize and value teaching and clinical care as a vital component of the academic mission. Academic anesthesiology research is threatened in the current climate, emphasizing production as reimbursements decline. In one institution, a new reimbursement system has been implemented, providing a significant increase in pay for clinical work over academic components, which further incentivizes non-research activities for those without external funding at the federal cap. There is a focus on being more technology-oriented, working on projects outside anesthesiology (aligned with current interests), collaborating more with non-anesthesiologist clinicians, and embracing more basic science research and less humanities-based work [10]. Some quotes depicting this phenomenon include the following. 

A shift to industry: one individual is "retiring from clinical medicine to focus solely on consulting within the industry."

Eat what you kill model: "At one place, a new 'eat what you kill' reimbursement system has been implemented, providing a significant increase in pay for clinical work over academic components, which further incentivizes non-research activities for those without external funding at the NIH cap."

Impact on next generation: "The lack of funding opportunities and senior mentorship is primarily affecting the next generation of researchers, making the path to an academic/research career very steep."

Concerns raised by some respondents in our sample focused on how the lack of funding opportunities and senior mentorship is primarily affecting the next generation of researchers, making the path to an academic/research career very steep. Further research needs to address leadership and mentorship, and how the next generation can receive the same level of support as prior generations of academic physicians. This is significant, as it is widely known that anesthesiologists are a specialty with a nearly a 50% burnout rate across the board. Burnout can dramatically impact junior faculty starting their careers. Two respondents mentioned leaving academic or research careers.

Opportunities, limitations, and recommendations

Advocacy for federal research stability is critical to preserving the momentum of anesthesiology research. Respondents emphasized the need for institutional bridge funding, protected research time, strengthened mentorship structures, and diversified funding sources, such as the Patient-Centered Outcomes Research Institute (PCORI) [11] and industry partnerships. The success of the Early-Stage Anesthesiology Scholars (eSAS) Program [12] serves as an excellent example, a grassroots initiative that has thrived well beyond expectations and demonstrates how peer-driven support can meaningfully advance academic anesthesiology. Similarly, the impact of the FAER Mentored Research Training Grant (MRTG) [13] highlights how targeted early-career funding can catalyze research productivity and career development, further underscoring the importance of expanding philanthropic and institutional investment in the field. There is a need to pursue more robust funding from charities and philanthropic benefactors, an effort that should be proactively led by departmental and institutional leadership, as in other fields of academia. Despite widespread concerns about research funding, 32 respondents (73%) stated that funding limitations did not affect their promotion or career trajectory. The current financial climate across many academic medical centers and universities, marked by tightening operational budgets and shifting institutional priorities, has substantially reduced discretionary support for research. As a result, mechanisms such as bridge funding, seed grants, and protected time have become increasingly scarce, further constraining the ability of clinician-scientists to sustain academic trajectories during funding gaps.

A follow-up survey aiming for a more robust response rate across other academic fields would help in understanding the overall impact of changing economic forces on academic medicine. Willingness to support starter grants and changes in core facilities likely influenced career satisfaction and burnout. Funding reductions (27%) may have contributed to stress indicators, but positive coping scores suggested adaptive strategies and resilience [14-16]. A majority of respondents are full professors, with nearly a quarter having tenure. Full professor rank physicians with established research programs and experience might have more resources or protected time for research activities than lower rank faculty. We will follow up this study with a survey specifically examining rank and responses.

## Conclusions

This survey reveals a research community committed to scholarly advancement yet increasingly burdened by structural and financial pressures. These are preliminary insights from a limited sample that highlight areas of concern warranting confirmation by larger, more representative studies. Overall, the changes reflect a move toward more reliable funding sources (industry, foundation grants, etc.), an adaptation to current funding landscapes by shifting away from certain research areas like humanities work, and a strategic adjustment in research methods and collaborations. Taken together, these results highlight the need for sustained investment and structural support to ensure that anesthesiology continues to advance scientifically and meet future patient care demands. Targeted institutional and national strategies are essential to safeguard the future anesthesiology research workforce and maintain innovation. A lot is on the line.

## Appendices

Survey

Title: Impact of 2025 Change in Federal Funding priorities on Academic Anesthesiologists

Form Description: We are studying the impact of the 2025 federal funding reduction/ cuts on research activities of clinician scientists and academic anesthesiologists. Your participation is voluntary, anonymous, and greatly appreciated. We are not only interested in federal grants but all types of research funding.

Sections and Questions

Section 1: Demographics

1. What is your current role? (single)

Assistant Professor

Associate Professor

Full Professor

Clinical Instructor

Research Scientist

Other: [Short Answer]

2. How many years have you been practicing as an academic anesthesiologist or intensivist? (single)

<5 years

5-10 years

11-20 years

>20 years

3. What type of institution do you currently work in? (single)

University-affiliated hospital

Public academic Institution

Private academic Institution

Other: [Short Answer]

4. In which region are you located- specify State? (single)

Northeast U.S.-------

Midwest U.S.--------

South U.S.--------

West U.S.-------

Outside the U.S.--------

5. What type of funding (current or prior) did/do you hold? (multiple)

NIH (National Institutes of Health)

AHRQ (Agency for Healthcare Research and Quality)

AHA

DoD (Department of Defense)

PCORI

Private foundations (e.g. FAER, SCCM, IARS)

Industry sponsored 

Internal departmental funding

Other: [Short Answer]

6. Have you ever been funded through FAER? (single)

o   Yes

o   No

Section 2: Research Funding and Activities

7. Prior to recent funding reductions, what percentage of your professional time was protected for research? (single)

0-10%

11-25%

26-50%

51-75%

>75%

8. How much protected time for research do you have now?

0-10%

11-25%

26-50%

51-75%

>75%

9. Have you been involved in a multi-institutional study?  (single)

Yes

No

10. If yes: were/are you the overall PI?  (single)

Yes

No

11. Have you experienced a reduction in research funding over the past 1 year? (single)

Yes

No

12. If yes, what was the primary source of funding that was reduced? (multiple)

NIH (National Institutes of Health)

AHRQ (Agency for Healthcare Research and Quality)

AHA

DoD (Department of Defense)

13. Were these cuts instituted by: (multiple)

·      External funders

·      Institution

·      Department

·      Self

·      Other

14. Have funding changes impacted your ability to: (multiple)

Conduct research

Publish manuscripts

Mentor trainees in research

Submit grant applications

Contribute to institutional indirect funding and research infrastructure

Other---------

15. Have you had to reduce or terminate any research projects due to funding cuts? (single)

Yes

No

·       Not applicable

16. If you have continued research, have you had to abandon elements of the trial (e.g. costly tests, or slow down recruitment)? (single)

Yes

No

Section 3: Clinical and Academic Responsibilities

17. As a result of reduced research funding, have your clinical duties changed? (single)

Increased clinical load

No change

Decreased clinical load

Other: [Short Answer]

18. If you have had a reduction in non-clinical time, will this impact your ability to execute research studies? (single)

Yes

No

19. Have you had to lay-off research related staff (technicians, research coordinators etc.)?  (single)

Yes

No

20. Has your academic promotion or career advancement been affected by funding reductions? (single)

Significantly delayed

Mildly delayed

No impact

Accelerated

Not applicable

Section 4: Well-Being and Future Research Plans

21. To what extent has the funding situation affected the following? (multiple with Likert scale)

Job satisfaction

Burnout symptoms

Career plans

22. What are your future plans regarding research involvement? (single)

Plan to leave research entirely

Plan to decrease research involvement

Plan to continue current level of research 

Plan to increase research involvement

23. What alternative funding platforms have you used? (multiple)

o   Foundation grants

o   Other public funding: specify

o   Foreign grants

o   Non profit organisations

o   Philanthropic organisations

24. Did you change/ modify the focus of your research/ projects? If yes, how?(single)

o   Yes

o   No

(Paragraph text) 

Section 5: Perceived Stress SurveyÒ [Free for non profit use]

25. In the last month, how often have you... (single)

(1-Never 2-Almost 3-Never 4-Sometimes 5-Fairly 6-Often 7-Very 8-Often)

1.     been upset because of something that happened unexpectedly? 0 1 2 3 4

2.     felt that you were unable to control the important things in your life? 0 1 2 3 4

3.     felt nervous and "stressed"? 0 1 2 3 4

4.     felt confident about your ability to handle your personal problems? 4 3 2 1 0

5.     felt that things were going your way? 4 3 2 1 0

6.     found that you could not cope with all the things that you had to do? 0 1 2 3 4

7.     been able to control irritations in your life? 4 3 2 1 0

8.     felt that you were on top of things? 4 3 2 1 0

9.     been angered because of things that were outside of your control? 0 1 2 3 4

10.  felt difficulties were piling up so high that you could not overcome them? 0 1 2 3 4

Section 6: Open Comments

23. Please provide any additional comments about how funding reduction have impacted your work, department, or field: (Paragraph text)

24. Potential suggestions? (multiple)

·      Diversify Funding Sources- Private or foundation

·      Focus on Health Services & Outcomes Research- database 

·      Build Collaborations- with other organisations to build on existing funding

·      Align with NIH Priorities

·      Leverage Innovation- Use of AI and other technology

·      Maximize Visibility- Publish and present in meetings

·      Engage in Advocacy- Join ASA etc in advocating for research

·      Strategic Career Moves- move to other tracks like education

·      Other (free text)
